# Risk of future trauma based on alcohol screening scores: A two-year prospective cohort study among US veterans

**DOI:** 10.1186/1940-0640-7-6

**Published:** 2012-04-30

**Authors:** Alex H S Harris, Anna Lembke, Patricia Henderson, Shalini Gupta, Rudolf Moos, Katharine A Bradley

**Affiliations:** 1 Center for Health Care Evaluation, VA Palo Alto Health Care System, 795 Willow Road, Menlo Park, CA, 94025, USA; 2 Group Health Research Institute, and Health Services Research & Development (HSR&D) Northwest Center of Excellence, Center of Excellence in Substance Abuse Treatment and Education (CESATE), Veterans Affairs (VA) Puget Sound Health Care System, 1100 Olive Way, 14th Floor, Seattle, WA, 98101, USA; 3 Departments of Medicine and Health Services, University of Washington, 1959 NE Pacific Street, Seattle, WA, 98195, USA

**Keywords:** Alcohol, Trauma, Fracture, AUDIT-C, Age, Gender, Screening, Women

## Abstract

**Background:**

Severe alcohol misuse as measured by the Alcohol Use Disorders Identification Test–Consumption (AUDIT-C) is associated with increased risk of future fractures and trauma-related hospitalizations. This study examined the association between AUDIT-C scores and two-year risk of any type of trauma among US Veterans Health Administration (VHA) patients and assessed whether risk varied by age or gender.

**Methods:**

Outpatients (215, 924 male and 9168 female) who returned mailed AUDIT-C questionnaires were followed for 24 months in the medical record for any International Statistical Classification of Diseases and Related Health Problems (ICD-9) code related to trauma. The two-year prevalence of trauma was examined as a function of AUDIT-C scores, with low-level drinking (AUDIT-C 1–4) as the reference group. Men and women were examined separately, and age-stratified analyses were performed.

**Results:**

Having an AUDIT-C score of 9–12 (indicating severe alcohol misuse) was associated with increased risk for trauma. Mean (SD) ages for men and women were 68.2 (11.5) and 57.2 (15.8), respectively. Age-stratified analyses showed that, for men ≤50 years, those with AUDIT-C scores ≥9 had an increased risk for trauma compared with those with AUDIT-C scores in the 1–4 range (adjusted prevalence, 25.7% versus 20.8%, respectively; OR = 1.24; 95% confidence interval [CI], 1.03–1.50). For men ≥65 years with average comorbidity and education, those with AUDIT-C scores of 5–8 (adjusted prevalence, 7.9% versus 7.4%; OR = 1.16; 95% CI, 1.02–1.31) and 9–12 (adjusted prevalence 11.1% versus 7.4%; OR = 1.68; 95% CI, 1.30–2.17) were at significantly increased risk for trauma compared with men ≥65 years in the reference group. Higher AUDIT-C scores were not associated with increased risk of trauma among women.

**Conclusions:**

Men with severe alcohol misuse (AUDIT-C 9–12) demonstrate an increased risk of trauma. Men ≥65 showed an increased risk for trauma at all levels of alcohol misuse (AUDIT-C 5–8 and 9–12). These findings may be used as part of an evidence-based brief intervention for alcohol use disorders. More research is needed to understand the relationship between AUDIT-C scores and risk of trauma in women.

## Background

Trauma is the leading cause of death in the United States in persons aged ≤44 years, and alcohol misuse is linked to higher risk of traumatic injury [1]. Alcohol is a factor in 60% of fatal burns, 40% of motor vehicle accidents with serious injury, and 42% of pedestrian fatalities [2]. In one study of 1118 adult patients admitted to trauma centers, 54% had a lifetime history of a substance use disorder, and 24% had a current diagnosis of alcohol dependence [3]. The annual cost of alcohol-related motor-vehicle accidents alone is US $51 billion [2].

Risk of future trauma may differ between individuals based not only on the amount of alcohol consumed but also on age and gender. Klatsky and Armstrong [4] found that people who drank six or more alcoholic drinks daily doubled their risk of death from motor-vehicle accidents, with women and those <50 years at especially high risk. Cherpitel et al. [5] found that risk of future trauma increased when consumption increased by as little as one standard daily drink in both men and women.

The Alcohol Use Disorders Identification Test–Consumption (AUDIT-C) is becoming an increasingly relied upon screening tool for alcohol use problems in modern medical practice [6]. It consists of only three questions, takes less than five minutes to administer, and has been validated across diverse medical settings [7-10]. The three AUDIT-C questions ask about the frequency of drinking, typical quantity of drinking, and the frequency of drinking six or more drinks in the past year. Higher AUDIT-C scores have been linked to future risk of negative outcomes including medication nonadherence [11], gastrointestinal illness [12,13], and all-cause mortality [14]. The data on AUDIT-C scores and future health risks provide relevant information for evidence-based discussions of alcohol misuse [15], part of a growing movement in primary care settings for improving screening, brief intervention, and referral to treatment (SBIRT) [16].

Scores on the AUDIT-C have been associated with risk of future trauma in two prior studies of US veterans. Harris et al. [17] found that severe alcohol misuse (AUDIT-C ≥8) was related to increased two-year risk of fractures. Williams et al. [18] demonstrated that severe alcohol misuse (AUDIT-C ≥8) was related to increased two-year risk of trauma-related hospitalization, including fractures, dislocations, lacerations, concussions, amputations, contusions, and burns.

This study builds on existing research on the relationship between AUDIT-C scores and risk of future trauma. Unlike prior work, which evaluated the risk of fracture and trauma-related hospitalization, this study examines AUDIT-C scores in relation to two-year risk of any type of trauma across all inpatient, residential, and outpatient settings in a large integrated health-care system. Also, the present study is an effort to verify the results of previous studies in a much larger sample with a specific focus on the differential risks associated with age and gender. The overarching goal is to provide more comprehensive sex- and age-tailored data for health-related discussions of alcohol misuse and risk of future trauma. Giving patients specific and tailored feedback about alcohol consumption has been shown to reduce hazardous alcohol use [19-21] and reduce alcohol related injuries by as much as 27% [22].

## Methods

### Data sources

AUDIT-C and covariable data were collected by the US Veterans Health Administration (VHA) as part of the population-based Survey of Healthcare Experiences of Patients (SHEP). The sampling strategy and logistics of the SHEP are described elsewhere [23]. Institutional Review Board (IRB) approval was obtained through the VHA and Stanford University for use of pre-existing administrative, clinical, and survey data. All statistical analyses were completed with SAS® statistical software, version 9.2 [24].

The AUDIT-C consists of the following three questions: (1) How often did you have a drink containing alcohol in the past year? (2) How many drinks containing alcohol did you have on a typical day when you were drinking in the past year? (3) How often did you have six or more drinks on one occasion in the past year? Each response is scored 0–4, with total AUDIT-C scores ranging from 0–12 [25]. Based on recent studies [11] and on evidence that low-level drinkers have lower risk of death and other medical problems than nondrinkers [12-14,26], we categorized AUDIT-C scores into four groups: 0, 1–4, 5–8, and 9–12, with 1–4 as the reference group *a priori*. Patients with AUDIT-C scores of 0 were past-year nondrinkers. Patients with AUDIT-C scores of 1–4 were considered low-level drinkers in this study. Patients with AUDIT-C scores 5–8 were considered positive for moderate alcohol misuse. Patients with AUDIT-C scores of ≥9 were considered positive for severe alcohol misuse (e.g., they reported drinking four or more times a week, drinking five drinks on a typical drinking day, and drinking six or more drinks on multiple occasions per week). One standard drink per the US National Institute on Alcohol Abuse and Alcoholism (NIAAA) is 12 ounces of beer, five ounces of wine, or 1.5 ounces of hard liquor [27], and patients were informed of these standards as part of SHEP.

### Sample

Patients who received ambulatory care in Veterans Affairs (VA) health-care facilities in 2004 and 2005, and who had not been surveyed in the past 12 months, were eligible for the SHEP in the month after an outpatient visit. Respondents’ International Statistical Classification of Diseases and Related Health Problems (ICD-9) diagnoses were obtained for VA health services received in the two years following the survey. Respondents had to have used VA health services at least once during the two-year follow-up period to be eligible. Utilization was assessed across diverse treatment settings in a large integrated health care system, including inpatient (e.g., medical, surgical, psychiatric), residential (e.g., mental health, nursing home), outpatient (e.g., primary care), and emergency departments.

**Figure 1:**
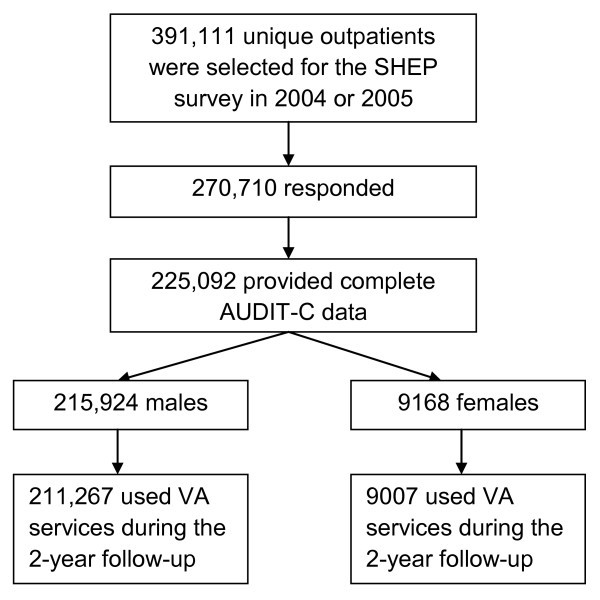
Patient sampling, response, and inclusion.

Figure 1 presents the flow of patient sampling, response, and inclusion. Overall, 391,111 patients were selected for the SHEP survey, and 270,710 responded (69%). From the sample of responders, 225,092 provided usable AUDIT-C data (i.e., they answered all three questions), 215,924 of whom were men and 9168 of whom were women. Of these, 211,267 men and 9007 women used VA health services at least once during the two-year follow-up period and, therefore, comprised the main analysis sample. Response rates were somewhat higher for men than for women and for patients ≥50 years than for those who were younger [28].

### Outcome measures

Outcomes included all clinically recorded ICD-9 diagnoses for trauma in the two years after completing the AUDIT-C. Trauma was defined as any wounds, amputations, musculoskeletal injuries, fractures (osteoporotic or nonosteoporotic), spinal cord injuries, burns, complications of trauma, poisoning, external causes of injury (e.g., severe sunburn, electrocution), concussions and brain injuries, internal injuries, and abuse (i.e., an injury inflicted by another person). (A list ICD-9 codes used to define trauma is available from the authors.) The primary outcome was an indicator (0 or 1) signifying whether the patient experienced any form of trauma, as defined above, during the two-year observation period. Analyses were conducted on each subtype of trauma (e.g., wounds, amputations) to determine if the overall pattern for all traumas obscured underlying differences.

### Covariables

The SHEP included questions about race, education (less than high school, high school graduate, college graduate), married status (no/yes), and cigarette smoking status (current, past year, 1–5 years ago, over five years ago, never). Age was obtained from the VA National Patient Care Database (NPCD). The Deyo comorbidity index, adapted from the Charleston Index for use with ICD-9 administrative data, was constructed from the NPCD based on inpatient and outpatient ICD-9 diagnostic codes assigned to participants in the year prior to taking the SHEP [29].

### Analysis strategy

Initial analyses described the sample and calculated the unadjusted prevalence of the outcomes in each AUDIT-C group. Then, covariable-adjusted logistic regression models were used to assess the risk of each outcome based on AUDIT-C risk group. *A priori* AUDIT-C group by gender and age interaction effects were evaluated. The interaction of gender and AUDIT-C group was marginally significant (p = 0.06). Given this interaction and the lack of data evaluating the association between alcohol screening scores and health outcomes in women, we stratified all subsequent analyses by gender. Because the recommended alcohol consumption limits for women are lower than those for men, and their recommended threshold for a positive AUDIT-C score is ≥2 compared with ≥3 for men, we examined different AUDIT-C groupings (score of 1–2 for the reference group, with 0, 3–4, 5–8 and 9–12 as comparators) for women in sensitivity analyses. We also performed age-stratified analyses in men because significant interactions were found, and because prior studies of AUDIT-C and health outcomes suggested age-moderated risk [13,14].

## Results

### Sample characteristics and distribution of AUDIT-C scores

Tables 1 and 2 present the characteristics of the male and female samples by AUDIT-C group, respectively. Of note, women were much younger and had lower levels of comorbidity than men. The mean (SD) ages of the male and female samples were 68.2 (11.5) and 57.2 (15.8) years, respectively. The majority of men and women were Caucasian (85% and 75%, respectively), and finished high school but not college (63% and 65%, respectively). Sixty-eight percent of men were married compared with 39% of women. Of the men and women in the sample, 44.8% and 48.0%, respectively, had an AUDIT-C score of 0; 42.0% and 46.5%, respectively, had an AUDIT-C score of 1–4; 10.5% and 4.4%, respectively, had an AUDIT-C score of 5–8; and 2.7% and 1.1%, respectively, had an AUDIT-C score of 9–12. Greater proportions of men, unmarried patients, current smokers, and Caucasian patients were represented in the AUDIT-C 9–12 group. In both the male and female samples, the AUDIT-C = 0 group had the highest levels of comorbidity.

**Table 1:** Characteristics of male outpatients in study sample (n = 211,267) by AUDIT-C score

		**AUDIT-C Score**			
	**0**		**1–4**	**5–8**	**9–12**
	**(94,620)**		**(88,790)**	**(22,103)**	**(5754)**
	N (Col %)		N (Col %)	N (Col %)	N (Col %)
Age	<50	4400 (4.65)	5236 (5.90)	2027 (9.17)	694 (12.1)
	50–65	28,377 (30.0)	26,045 (29.3)	10,044 (45.4)	3400 (59.1)
	>65	61,841 (65.4)	57,508 (64.8)	10,032 (45.4)	1660 (28.8)
Race/ethnicity	White	78,923 (83.4)	77,131 (86.9)	18,140 (82.1)	4506 (78.3)
	African American	6323 (6.68)	4405 (4.96)	1638 (7.41)	473 (8.22)
	Hispanic	4134 (4.37)	3368 (3.79)	1318 (5.96)	475 (8.26)
	Other	3366 (3.56)	2344 (2.64)	691 (3.13)	220 (3.82)
Education	<12^th^ grade	23,297 (24.6)	11,834 (13.3)	3051 (13.8)	898 (15.6)
	High school graduate	57,529 (60.8)	55,526 (62.5)	14,894 (67.4)	4097 (71.2)
	College graduate	11,706 (12.4)	20,000 (22.5)	3872 (17.5)	677 (11.8)
Married		64,386 (68.0)	62,433 (70.3)	12,696 (57.4)	2624 (45.6)
Cigarette smoking	Never	26,714 (28.2)	23,296 (26.2)	4595 (20.8)	1155 (20.1)
	Past year	4352 (4.60)	4066 (4.58)	1724 (7.80)	524 (9.11)
	1–5 years ago	4735 (5.00)	3826 (4.31)	1183 (5.35)	321 (5.58)
	>5 years ago	48,473 (51.2)	48,496 (54.6)	10,015 (45.3)	1846 (32.1)
	Current	10,346 (10.9)	9106 (10.3)	4586 (20.7)	1908 (33.2)
Deyo comorbidity index score,* mean (SD)		0.97 (1.04)	0.72 (.91)	0.59 (.81)	0.57 (.79)

*Adapted from the Charleston index for use with ICD-9 administrative data and constructed from the NPCD based on patients’ past-year inpatient and outpatient ICD-9 diagnostic codes.

**Table 2:** Characteristics of female outpatients (n = 9007) by AUDIT-C score

		**AUDIT-C Score**			
	**0**		**1–4**	**5–8**	**9–12**
	**(4319)**		**(4191)**	**(400)**	**(97)**
	**N (Col %)**		**N (Col %)**	**N (Col %)**	**N (Col %)**
Age	<50	1157 (26.8)	1679 (40.1)	203 (50.8)	63 (64.9)
	50–65	1681 (38.9)	1413 (33.7)	135 (33.8)	30 (30.9)
	>65	1481 (34.3)	1099 (26.2)	62 (15.5)	4 (4.12)
Race/ethnicity	White	3212 (74.4)	3228 (77.0)	288 (72.0)	61 (62.9)
	African American	536 (12.4)	461 (11.0)	68 (17.0)	19 (19.6)
	Hispanic	202 (4.68)	208 (4.96)	17 (4.25)	7 (7.22)
	Other	267 (6.18)	228 (5.44)	18 (4.50)	6 (6.19)
Education	<12^th^ grade	198 (4.58)	62 (1.48)	11 (2.75)	2 (2.06)
	High school graduate	2935 (68.0)	2591 (61.8)	278 (69.5)	61 (62.9)
	College graduate	1102 (25.5)	1493 (35.6)	104 (26.0)	31 (32.0)
Married		1671 (38.7)	1666 (39.8)	130 (32.5)	25 (25.8)
Cigarette smoking	Never	2009 (46.5)	1752 (41.8)	130 (32.5)	32 (33.0)
	Past year	222 (5.14)	265 (6.32)	43 (10.8)	16 (16.5)
	1–5 years ago	197 (4.56)	244 (5.82)	30 (7.50)	2 (2.06)
	>5 years ago	1189 (27.5)	1255 (29.9)	65 (16.3)	7 (7.22)
	Current	702 (16.3)	675 (16.1)	132 (33.0)	40 (41.2)
Deyo comorbidity index score,* mean (SD)		0.69 (.91)	0.43 (.71)	0.30 (.59)	0.31 (.57)

*Adapted from the Charleston index for use with ICD-9 administrative data and constructed from the NPCD based on patients’ past-year inpatient and outpatient ICD-9 diagnostic codes.

### AUDIT-C risk group and trauma in men

Table 3 presents the unadjusted cross-tabulation of AUDIT-C categories and the two-year prevalence of trauma outcomes for men. The two-year prevalence of any trauma was 11.6%, with 10.5% of patients in the AUDIT-C 1–4 group experiencing trauma over two-year follow-up compared with 15.4% in the AUDIT-C 9–12 group. The AUDIT-C 9–12 group had the highest unadjusted prevalence of most types of trauma, especially the more common types. In examining the association between AUDIT-C risk group and two-year prevalence of trauma for men, several significant interactions between risk groups and covariables were found, including an interaction between AUDIT-C risk group and age (p = 0.02). To clarify these associations, we stratified the male sample into three age groups (<50, 50–65, and >65) and re-ran the adjusted analyses (Table 4).

**Table 3:** Unadjusted frequency of trauma in men by AUDIT-C score

**Outcome**	**AUDIT-C Score**				**2-Year Prevalence (%)**
	**0**	**1–4**	**5–8**	**9–12**	
	**(94,620)**	**(88,790)**	**(22,103)**	**(5754)**	
Any kind of trauma	11,542 (12.2)	9311 (10.5)	2762 (12.5)	887 (15.4)	11.60
Wounds and amputations	6277 (6.63)	4846 (5.46)	1345 (6.09)	415 (7.21)	6.10
Musculoskeletal injuries	3577 (3.78)	3201 (3.61)	995 (4.50)	276 (4.80)	3.81
Fractures	2224 (2.35)	1733 (1.95)	601 (2.72)	252 (4.38)	2.28
Fracture: nonosteoporotic	1616 (1.71)	1331 (1.50)	458 (2.07)	189 (3.28)	1.70
Fracture: osteoporotic	810 (0.86)	553 (0.62)	197 (0.89)	88 (1.53)	0.78
Nonfracture trauma	10,134 (10.7)	8199 (9.23)	2381 (10.8)	721 (12.5)	10.15
Spinal cord injuries, nerves	342 (0.36)	276 (0.31)	67 (0.30)	27 (0.47)	0.34
Burns	279 (0.29)	177 (0.20)	56 (0.25)	16 (0.28)	0.25
Complications of trauma	226 (0.24)	178 (0.20)	54 (0.24)	21 (0.36)	0.23
Poisoning	186 (0.20)	159 (0.18)	55 (0.25)	11 (0.19)	0.19
External causes	167 (0.18)	131 (0.15)	34 (0.15)	7 (0.12)	0.16
Concussions, brain injury	170 (0.18)	118 (0.13)	30 (0.14)	8 (0.14)	0.15
Internal injuries	107 (0.11)	74 (0.08)	30 (0.14)	11 (0.19)	0.11
Physical abuse	48 (0.05)	20 (0.02)	4 (0.02)	7 (0.12)	0.04

**Table 4:** Two-year adjusted prevalence of all trauma, fractures only, and nonfracture trauma in men

	**AUDIT-C Score**			
	**0**	**1–4**	**5–8**	**9–12**
	2-Year Adjusted Rate*	2-Year Adjusted Rate*	2-Year Adjusted Rate*	2-Year Adjusted Rate*
**All Trauma**				
** Men <50**	0.221 (0.028)	0.208 (.027)	0.203 (0.037)	0.257 (0.050) ^†^
** Men 50–65**	0.169 (0.037) ^†^	0.151 (0.038)	0.154 (0.035)	0.156 (0.040)
Men >65**	0.093 (0.032) ^†^	0.074 (0.029)	0.079 (0.030) ^†^	0.111 (0.034) ^†^
**Fractures Only**				
** Men <50**	0.041 (0.013)	0.036 (0.011)	0.046 (0.016)	0.067 (0.025) ^†^
** Men 50–65**	0.030 (0.010)	0.027 (0.011)	0.033 (0.013)	0.044 (0.020) ^†^
Men >65**	0.019 (0.010) ^†^	0.014 (0.008)	0.018 (0.009) ^†^	0.034 (0.018) ^†^
**Nonfracture Trauma**				
** Men <50**	0.201 (0.026)	0.186 (0.022)	0.175 (0.035)	0.216 (0.049)
** Men 50–65**	0.151 (0.033) ^†^	0.135 (0.033)	0.134 (0.030)	0.128 (0.029) ^†^
Men >65**	0.080 (0.027)^†^	0.064 (0.025)	0.067 (0.026)	0.086 (0.036)^†^

*Prevalence (SD) from multivariable logistic regression models adjusted for age, race, education, marital status, cigarette smoking status, and Deyo comorbidity index scores.

**Significant interactions with AUDIT-C scores and education and comorbidity were found in men >65 years.

^†^Significantly different from reference group adjusted in multivariable logistic regression models (p < 0.05).

### AUDIT-C risk group and trauma in men: Age-stratified analyses

For men aged <50 years, those with AUDIT-C scores of ≥9 (indicating severe misuse) had an increased risk for trauma compared with those with AUDIT-C scores in the 1–4 range (adjusted prevalence, 25.7% versus 20.8%; OR = 1.24, 95% CI: 1.03–1.50). Covariables associated with increased risk of trauma were Caucasian race/ethnicity, being single, having more comorbid conditions, and being a current or past smoker. No significant interactions between AUDIT-C group and covariables were found in this subgroup.

For men aged 50–65 years, only those with AUDIT-C scores of 0 (past-year nondrinker) had an increased risk for trauma compared with those with AUDIT-C scores in the 1–4 range (adjusted prevalence, 16.9% versus15.1%; OR = 1.09, 95% CI: 1.04–1.14). In these middle-aged men, covariables associated with increased risk of trauma were non-Caucasian race/ethnicity, being single, having more comorbid conditions, and being a current or past smoker. No significant interactions between AUDIT-C group and covariables were found in this subgroup.

For men aged >65 years with average comorbidity and education, both those with AUDIT-C scores of 5–8 (adjusted prevalence, 7.9% versus 7.4%; OR = 1.16, 95% CI: 1.02–1.31) and ≥9 (adjusted prevalence, 11.1% versus 7.4%; OR = 1.68, 95% CI: 1.30–2.17) had significantly increased risk of trauma compared with the reference group. Nondrinkers aged ≥65 years were also at increased risk of trauma compared with those in the AUDIT-C 1–4 group (adjusted prevalence, 9.3% versus 7.4%; OR = 1.22, 95% CI: 1.14–1.31). In all men ≥65 years, significant interactions between AUDIT-C group, Deyo comorbidity index, and education were found: in general, compared with patients in the AUDIT-C 1–4 category, patients in the other categories were at greater risk for trauma if they had fewer comorbidities or more or less than a high-school education.

### AUDIT-C risk group in men and specific categories of trauma: Age-stratified analyses

Age-stratified analyses of specific types of trauma in men revealed similar patterns across age groups for fractures as for all other types of trauma combined (Table 4). Men aged <50 years with AUDIT-C scores ≥9 had a significantly increased risk of fractures (adjusted prevalence, 6.7% versus 3.6%; OR = 1.74, 95% CI: 1.24–2.44). Men aged 50–65 years who had AUDIT-C scores ≥9 had a significantly increased risk of fractures (adjusted prevalence, 4.4% versus 2.7%; OR = 1.39, 95% CI: 1.16–1.68). Men aged >65 years with AUDIT-C scores of 5–8 (adjusted prevalence, 1.8% versus 1.4%; OR =1.52, 95% CI: 1.18–1.98) and 9–12 (adjusted prevalence, 3.4% versus 1.4%; OR = 2.75, 95% CI: 1.74–4.32) had significantly increased risk of fractures. No other specific type of trauma was significantly associated with AUDIT-C scores in stratified analyses. When all nonfracture traumas were combined, men aged >65 years in the AUDIT-C 9–12 group had a significantly higher prevalence (8.6%) compared with men in the AUDIT-C 1–4 (reference) group (6.4%). However, men in the 50–65 age group with AUDIT-C scores of 9–12 had significantly lower prevalence (12.8%) compared with men in the AUDIT-C 1–4 (reference) group (13.5%).

### AUDIT-C risk group and trauma in women

No association between AUDIT-C scores and trauma outcomes was found among women (Table 5). Sensitivity analyses examined alternative AUDIT-C groupings (score of 1–2 as the reference group with 0, 3–4, 5–8 and 9–12 as comparators) and the single item about frequency of heavy drinking (≥six drinks on one occasion) as a predictor. No associations were found in these analyses.

**Table 5:** Unadjusted frequency of trauma in women by AUDIT-C score

	**AUDIT-C Score**				
**Outcome**	**0**	**1–4**	**5–8**	**9–12**	**2-Year Prevalence (%)**
	**(4319)**	**(4191)**	**(400)**	**(97)**	
All trauma	961 (22.3)	893 (21.3)	87 (21.8)	21 (21.6)	21.8
Wounds and amputations	494 (11.4)	405 (9.66)	41 (10.3)	10 (10.3)	10.5
Musculoskeletal injuries	402 (9.31)	406 (9.69)	45 (11.3)	10 (10.3)	9.6
Fractures	216 (5.00)	159 (3.79)	15 (3.75)	5 (5.15)	4.4
Fracture: nonosteoporotic	68 (1.57)	41 (0.98)	4 (1.00)	2 (2.06)	1.3
Fracture: osteoporotic	182 (4.21)	136 (3.25)	12 (3.00)	5 (5.15)	3.7
Nonfracture trauma	874 (20.2)	815 (19.4)	79 (19.8)	18 (18.6)	19.83
Spinal cord injuries, nerves	17 (0.39)	18 (0.43)	2 (0.50)	0	0.4
Burns	18 (0.42)	22 (0.52)	0	0	0.4
Complications of trauma	14 (0.32)	11 (0.26)	1 (0.25)	1 (1.03)	0.3
Poisoning	12 (0.28)	6 (0.14)	0	1 (1.03)	0.3
External causes	5 (0.12)	10 (0.24)	1 (0.25)	0	0.2%
Concussions, brain injury	13 (0.30)	7 (0.17)	1 (0.25)	1 (1.03)	0.3%
Internal injuries	13 (0.30)	3 (0.07)	0	0	0.2%
Physical abuse	69 (1.60)	67 (1.60)	4 (1.00)	1 (1.03)	1.6%

## Discussion

To our knowledge, this is the first study to examine the relationship between alcohol screening scores (AUDIT-C) and risk of inpatient, residential, and outpatient medical treatment for any type of trauma. This is also one of few studies to look at women’s trauma risk separate from men’s. In male VHA patients, severe alcohol misuse (AUDIT-C 9–12) was associated with a significantly increased risk for trauma, particularly fractures. Men in the oldest age group (>65 years) showed increased risk of medical care for trauma even at lower levels of alcohol misuse (AUDIT C 5–8). These findings augment prior research, which found an association between severe alcohol misuse and specific risk of fracture [17], and between severe alcohol misuse and trauma-related hospitalization [18].

The AUDIT-C was not a predictor of two-year trauma risk in female VHA patients. The absence of an association is counterintuitive, since women have been shown to be more vulnerable to the toxic effects of alcohol [30,31]. Other studies of AUDIT-C scores from the SHEP survey and subsequent health outcomes had similar findings, in that associations between AUDIT-C scores and new-onset liver disease, upper gastrointestinal bleeding, and pancreatitis in the subsequent two years was associated with AUDIT-C scores in men but not in women.

Several factors may be affecting the results on for women in the present study. First, increased stigma for heavy drinking among women typically leads to underreporting drinking levels compared with men [31,32]. Also, even with a sample of over 9000 women, the number of women with alcohol misuse was relatively small (400 with AUDIT-C scores of 5–8 and 97 with AUDIT-C scores of 9–12), and there were relatively small numbers of total traumas (21) and fractures (5) in the highest AUDIT-C group. In addition, it is possible that women who screened positive for alcohol misuse were less likely to have full VA benefits, which could lead to under-ascertainment of trauma in women with alcohol misuse compared with women without alcohol misuse. Furthermore, even though the survey was confidential, women may have under-reported alcohol use more frequently than men because, given their younger age, they might still have been applying for VA benefits (often from military sexual trauma), and may have worried that, if they accurately reported their drinking, they would be denied. However, these speculations do not explain results from another study with the same sample that found severe alcohol misuse (AUDIT-C 9–12) had an even more pronounced effect on mortality among women than among men [14]. Taken together, these results highlight the importance of sex-tailored risk information as well as then need for more research to clarify these relationships. Regardless, women who screen positive for alcohol misuse should be counseled to reduce their drinking and advised of the other documented medical risks (e.g., mortality) of at-risk consumption.

Fractures comprised the third leading cause of trauma for men (2.28%), exceeded only by wounds/amputations and musculoskeletal injuries. The association between alcohol misuse and fractures may be related to the increased risk of falls due to intoxication, with potential contribution from neurologic complications of alcohol misuse such as cerebellar degeneration, Wernicke-Korsakoff syndrome, peripheral neuropathy [33], and increased risk of motor-vehicle accidents [2]. Furthermore, alcohol misuse has been linked to the development of osteoporosis, which also increases the risk of fractures [34].

On average, older men (>65) had a heightened risk of trauma with lower levels of misuse (AUDIT-C 5–8) as well as severe misuse (AUDIT-C 9–12). This finding is consistent with two prior studies of AUDIT-C and future trauma risk [17,18], both of which showed lower consumption associated with elevated risk in older men. In our study, among men >65 years, those with severe alcohol misuse (AUDIT-C 9–12) had double the risk of fracture compared with same-aged men drinking in moderation (AUDIT-C 1–4). The heightened alcohol-related risk in older men may be related to a greater propensity to fall and a greater risk of fracture from a fall.

Nondrinkers aged >50 years were at increased risk for injuries and accidents over the two-year follow-up. Previous studies found that nondrinkers were at increased risk for adverse health outcomes, including fractures and primary or secondary trauma-related hospitalization [17,18] compared with people drinking at low levels. Nondrinkers were older, had greater morbidity, and had poorer health status, making them more susceptible to falls and resultant fractures. Many people reporting themselves as nondrinkers had likely stopped drinking due to medical problems that could contribute to accidents [35]. Furthermore, Holahan et al. [26] demonstrated that abstainers are more likely than moderate drinkers to have had prior drinking problems, to be obese, and to smoke cigarettes.

Although our data do not distinguish between former at-risk drinkers and lifetime nondrinkers, nondrinking men aged <50 years had no increased trauma risk. It is likely that, with age, some formerly heavy-drinking men migrate to the nondrinking group and potentially increase the trauma risk of nondrinkers. Consistent with this hypothesis, older nondrinking men had more comorbid illnesses than younger nondrinking men and older men with alcohol misuse. In other words, the increased risk of trauma among abstainers might be due, in part, to alcohol-related harm from former heavy drinking [26]. Given the consistency of this finding across studies, it would be important for future studies to distinguish between former problem drinkers and lifetime nondrinkers.

These results need to be understood in light of several limitations. The sample consisted exclusively of patients of the VHA, who are predominantly male and older than the general population. Only 69% of selected patients responded to the SHEP, and only 83% of those provided complete AUDIT-C data. Although we have no reason to expect that the relationship between AUDIT-C scores and subsequent trauma is different in the observed and unobserved samples, this possibility must be considered. Also, with samples this large, some (but certainly not all) of the statistically significant differences in risk were small in absolute and relative terms.

Also, misclassification and under-ascertainment of outcomes threaten the internal validity of this study. Outpatient ICD-9 codes are not as valid as inpatient codes [36]. Many patients in the VHA system use Medicare and do not necessarily get transferred to VA medical centers, which likely decreases the accurate detection of health-care utilization in the older age group [36]. Others have limited VA eligibility and may seek emergency care outside the VA health-care network. As described above, the AUDIT-C assesses drinking in the past year but cannot differentiate lifetime abstainers from previous high-risk or problem drinkers. Finally, AUDIT-C data collected in the course of a mailed patient-satisfaction survey may differ in important ways from screening data obtained in the course of clinical care [37]. Any of these limitations may have caused an over- or underestimation of the magnitude or direction of the association between AUDIT-C scores and trauma.

In summary, the AUDIT-C is a useful scaled marker of two-year risk of trauma across different treatment settings in men. Severe alcohol misuse is most strongly associated with risk of subsequent fracture in older men who are otherwise relatively healthy. Older men with moderate alcohol misuse are also at increased risk. We did not find an association between AUDIT-C scores and risk of trauma diagnoses in women, but we hypothesize that this had more to do with under-ascertainment bias than lack of an association between alcohol use and trauma in women. Our data add to the growing body of evidence in support of the AUDIT-C as a marker for future health risk among men, including trauma, medication nonadherence [11], gastrointestinal illness [12,13], and all-cause mortality [14].

## Competing interests

The authors declare that they have no competing interests.

## Authors’ contributions

AH conceived of the study, supervised the acquisition and analysis of data, and helped draft the manuscript. AL drafted the manuscript and assisted in the interpretation of the data. PH and SG led the statistical analyses. RM and KB helped conceive the study, helped with interpretation of the data, and helped draft the manuscript. All authors read and approved the final manuscript.
